# Examination of sulfonamide-based inhibitors of MMP3 using the conditioned media of invasive glioma cells

**DOI:** 10.1080/14756366.2020.1715387

**Published:** 2020-03-11

**Authors:** Alisha T. Poole, Christopher A. Sitko, Caitlin Le, Christian C. Naus, Bryan M. Hill, Eric A. C. Bushnell, Vincent C. Chen

**Affiliations:** a Department of Chemistry, Brandon University, Brandon, Canada; b Department of Cellular and Physiological Sciences, University of British Columbia, Life Science Institute, Vancouver, Canada

**Keywords:** Matrix metalloproteinase, glioblastoma multiforme, inhibition, ilomastat

## Abstract

Glioblastoma multiforme (GBM) is the deadliest and the most common primary malignant brain tumour. The median survival for patients with GBM is around one year due to the nature of glioma cells to diffusely invade that make the complete surgical resection of tumours difficult. Based upon the connexin43 (Cx43) model of glioma migration we have developed a computational framework to evaluate MMP inhibition in materials relevant to GBM. Using the ilomastat Leu-Trp backbone, we have synthesised novel sulphonamides and monitored the performance of these compounds in conditioned media expressing MMP3. From the results discussed herein we demonstrate the performance of sulfonamide based MMPIs included AP-3, AP-6, and AP-7.

## Introduction

Glioblastoma multiforme (GBM) is a malignant tumour of the brain^1^, accounting for more than half of all astrocytoma cases^2^. High-grade gliomas are characterised by proliferation, necrosis, angiogenesis, invasion, and evasion of apoptosis^3–5^. Despite radical treatment encompassing surgical resection, radiation and chemotherapy, the GBM patients have a poor outlook and a median survival of 10–15 months^6–9^. A major factor underlying the lethality of GBM, is the acquisition of therapeutic resistance and the presence of diffusely invading cells which renders the complete surgical resection difficult, if not impossible to achieve^10^^,^^11^.

Infiltration of cancer is reliant on the tumour microenvironment. In particular, the extracellular matrix (ECM) is an important regulator of cancer cell invasion, migration, and proliferation. Identification of genes that are differentially regulated by invasive glioma are of significant interest. Using a model of glioma migration invasion based on the gap junction protein connexin43 (Cx43)^12–17^ we recently demonstrated increases in the proteinase matrix metalloproteinase-3 (MMP3) within the conditioned media (secretome)^18^. These findings are supported by previous studies demonstrating MMP3 at invasive fronts of GBM tumours, and the reduction of invasion potential with MMP3 loss^19^. Signals mediated by MMPs include the activation/inactivation of growth factors, shedding of cell surface adhesion molecules, and ECM-bound cytokines, growth factors, and cryptic peptides^20^^,^^21^. As there is a strong correlation between patients’ outcomes with the activities of proteases within the extracellular space^22–27^, we sought to inhibit mechanisms that are linked to MMP3.

The importance of MMPs in cancer biology has led to the development of a large number of synthetic MMP inhibitors (MMPIs). Within the active site, a Zn^2+^ metal ion binds protein substrates that are marked for degradation. As a therapeutic strategy, the majority of MMPIs utilise a strong zinc-binding group (ZBG) to inactivate MMPs^28^. Despite a significant body of data implicating MMPs in the invasion of cancer, the clinical potential of MMPIs remains largely unfulfilled. Despite considerable promise, MMPIs have yet to pass clinical trial for the treatment of cancer^29^. The vast majority of MMPIs tested, including the broad-spectrum inhibitor ilomastat, utilise a hydroxamic acid as the ZBG^30–33^. A number of reasons for clinical trial failure have been proposed: (1) poor pharmacokinetics^34^; (2) poor transition metal/MMP selectively^35^; and (3) rapid metabolism^36^. Despite these confines, it is important to note selective inhibitors have proven to be less effective when applied to *in vivo* animal models^37^.

Despite a troubled history of hydroxamic acid-based MMPIs, very little attention has been paid towards the development of alternative ZBG^32^^,^^38–42^. Maintaining interests in MMPs as a therapeutic target, the broad-spectrum non-hydroxamic acid MMPI Periostat^TM^ (low dose doxycycline) is approved by the FDA for the treatment of periodontal disease^31^^,^^43^. Based on previous reports of ilomastat decreasing the invasiveness of high-grade astrocytoma via MMP3 inhibition^44^, and the known safety profiles of sulphonamide-based medicines (a.k.a. “sulfa” drugs)^45^^,^^46^, we sought to evaluate sulphonamides as novel ZBGs. Although the incorporation of the sulphonamides has been suggested to improve MMPIs by enhancing the molecule’s ability to form H-bonds^33^, the ability of the functional group to bind zinc and catalytically inactivate MMPs has not been sufficiently explored. To determine structure-activity relationships of sulfa-based inhibitors, we synthesised a small number of compounds based on the ilomastat (Leu-Trp) backbone to measure MMP3-inhibitory performance within matrices relevant to GBM^30^. Molecular docking and molecular dynamic studies revealed structural requirements to inhibit MMP3. Our datum suggests our sulphonamide/ZBG replacement strategy may have broad utility as future MMPIs.

## Materials and methods

### Compound synthesis

All starting materials and reagents were commercially available from Sigma-Aldrich and used without further purification. Reactions were monitored via TLC with 210–270 μm silica gel plates (EMD Chemicals Inc., 5715–7) using UV light and potassium permanganate. Flash column chromatography was performed using 230–400 mesh ultrapure silica gel (60 Å, Silicycle). Proton NMR of compound **D** was performed on a Bruker spectrometer (400 MHz) with DMSO-*d*6 as the solvent (Supplementary Figure S1). HPLC-MS was performed, analysis of the synthesised compounds by QTOF mass spectrometry (Agilent, 6530) (Supplementary Figure S2).

### Synthesis of compound B

**Figure 1.**
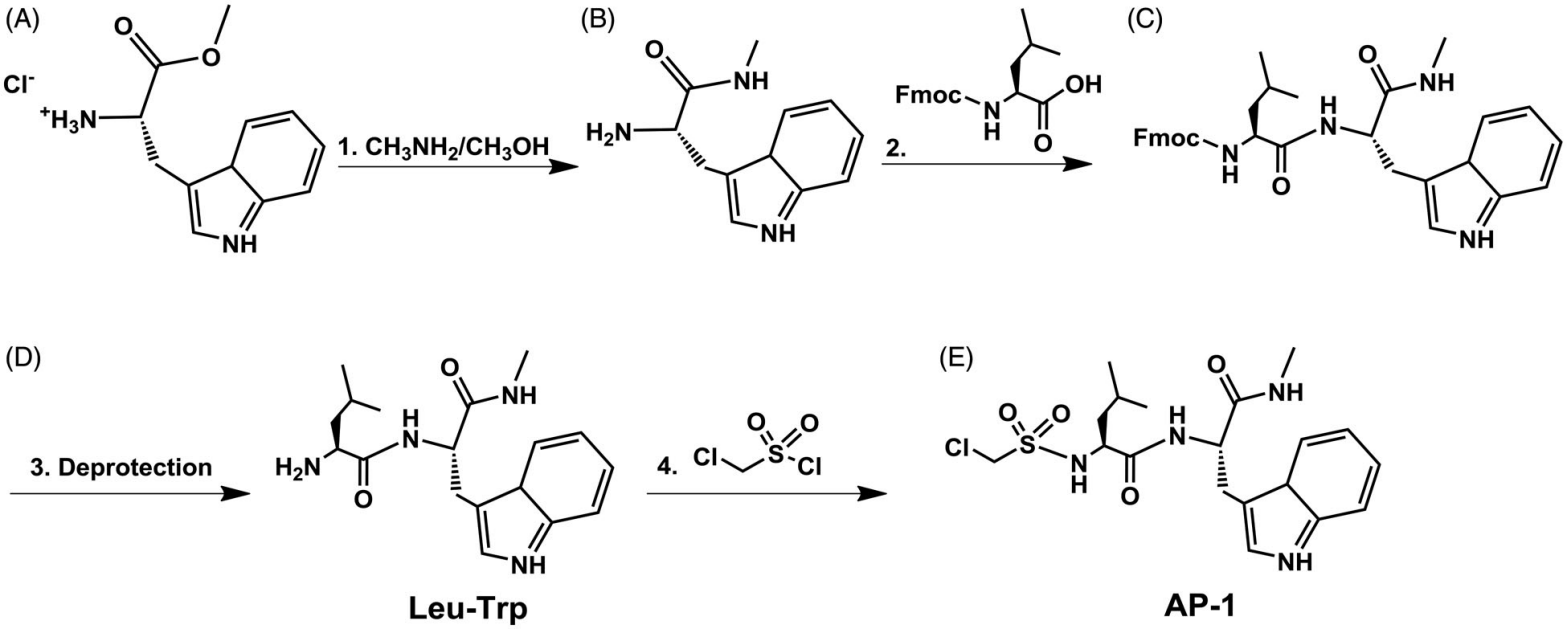
General synthesis scheme for derivatives: (1) **A** (1.0 eq.), CH_3_NH_2_/CH_3_OH (33% CH_3_NH_2_ by wt., 10 eq.); (2) **B** (1.0 eq.), FMOC-Leu-OH (2.0 eq.), DIC (2.0 eq.), Oxyma Pure (2.0 eq.), *N,N*-Diisopropylethylamine (2.0 eq.); (3) **C** (1.0 eq.), 20% piperidine in DMF; (4) **D** (1.0 eq.), chloromethane sulphonyl chloride (7.0 eq.), *N,N*-Diisopropylethylamine (7.0 eq.).

0.5062 g (1.963 mmol) L-tryptophan methyl ester hydrochloride (Figure 1A) in 2.5 ml of MeNH_2_/MeOH (33% MeNH_2_ by wt.) was mixed overnight under nitrogen. Product was then place on rotovap to evaporate MeOH and MeNH_2_ to yield L-tryptophan methyl amide (Figure 1B) as a yellow oil (0.4126 g, 95.6%).

### Synthesis of compound C

1.387 g (3.926 mmol) Fmoc-Leucine-OH was dissolved in 8 ml of 1:1 DMF:CH_2_Cl_2_ and mixed with 0.61 ml of DIC, 0.557 g of Oxyma Pure, and 0.70 ml of *N,N*-Diisopropylethylamine under nitrogen at room temperature for 10 min. The solution was then added to 0.4126 g (1.899 mmol) compound **B** and mixed for 48 h under nitrogen. Compound **C** was purified by flash column chromatography using 2:1 chloroform to methanol as the mobile phase. Fractions were collected, and solvent evaporated to yield a yellow-orange oil (0.8126 g, 77.4%). *Synthesis of Compound D (*Figure 1 Leu-Trp*):* 0.08126 g (0.147 mmol) FMOC-Leucine-Tryptophan was mixed with 4 ml of 20% piperidine in DMF for two hours. Compound **D** was purified by flash column chromatography using 2:1 chloroform to methanol as the mobile phase. Fractions were collected, and solvent evaporated to yield a yellow-orange oil (0.041 g, 83.9%, C_18_H_26_N_4_O_2,_ EM: 330.2055, LC-MS *m/z*: 331.217 (M + H), 353.195 (M + Na), NMR 400 MHz: ∼11 ppm H on N of Trp five carbon ring, ∼7 ppm Trp aromatic H, ∼1 ppm H on methyl groups of Leu).

### Synthesis of compound E (AP-1)

0.78 ml (8.627 mmol) of chloromethane sulphonyl chloride was added to 0.4097 g (1.23 mmol) of Compound **D** (Figure 1). 1.55 ml (8.627 mmol) of *N,N*-Diisopropylethylamine was then added and the mixture was refluxed under nitrogen overnight. Compound **E** (Figure 1) was purified by flash column chromatography using 2:1 chloroform to methanol as the mobile phase. Fractions were collected, and solvent evaporated to yield a reddish-orange oil (0.5154 g, 94.5%, C_19_H_27_ClN_4_O_4_S, EM: 442.1442, LC-MS *m/z*: 443.175 (M + H), 384.103 (M + H, metastable/in-source fragmentation, –C_2_H_5_NO).

### Computational methods

The 2016 version of MOE (Molecular Operating Environment) was used for all calculations and analysis discussed herein^47^. As a model the crystal structure of MMP-3 with co-crystalized NNGH (PDB: 4G9L) was used^48^. Using the Preparation Tool in MOE the alpha chain was deleted, hydrogens were added and protonation states of the ionisable amino acids were corrected. The protein was then solvated using a periodic boundary condition with NaCl counter ions added at a 0.1 mol/l concentration. The system was then energy minimised to a gradient of 0.1 kcal/mol Å^2^ where the Amber10:EHT force field was used. The minimised system was then ran for a 1.0 ns MD simulation using the NPA algorithm to allow the system to relax. A time step of 2 fs was used. Light bonds were constrained and rigid waters were used. Following the MD simulation the final structure where NNGH was deleted was used for all subsequent calculations.

For the docking a two-step approach was used; the first step involved the use of the triangle matcher placement method to generate random poses. Each pose was then scored using the London dG scoring function where the top 30 scoring poses were kept. In the second step, the 30 poses were then refined using the induced fit method in MOE and rescored using the Generalized-Born Volume Integral/Weighted Surface Area (GBVI/WSA) dG scoring function where the top five scoring conformers were kept.

The compounds docked are listed in Table 1. For the docking calculations the receptor was defined to be MMP-3 include the Zn^2+^ ion. As discussed in Jacobsen et al.^49^, favoured ZBGs are hydroxamic acids due to the formation of trigonal bipyrimidal coordination geometries around the Zn^2+^ ion. Moreover, the chelation of the hydroxamic acids to the Zn^2+^ ion enhances the acidity of the ZBG resulting in deprotonation of the OH group enhancing the binding of the ligands to the Zn^2+^ centre. The deprotonated OH group is further stabilised by forming a hydrogen bond interaction to the backbone neutral carboxylic side chain of Glu202 (numbering taken from PDB: 4G9L)^48^^,^^49^. Thus, for ilomastat, **AP-3**, **AP-4**, **AP-6**, and **AP-7** the acidic protons were removed prior to docking. For **Leu-Trp**, **AP-1**, **AP-2**, and **AP-5** no acidic protons are present. For all compounds Glu202 was modelled as neutral.

**Table 1.** The binding affinities and Zn coordination for ilomastat and various derivatives. R represents the Leu-Trp backbone of ilomastat.

Compound	Zinc Binding Group^a^	Score (kcal mol^−1^)
**Ilomastat**		−12.1
**Leu-Trp**		−8.3
**AP-1**		−7.7
**AP-2**		−8.6
**AP-3**		−11.2
**AP-4**		−9.6
**AP-5**		−9.3
**AP-6**		−12.3
**AP-7**		−11.6

^a^ R is the Leu-Trp backbone. See Figure 5(D) for the complete structure of compound.

For the ligands that had predicted Gibbs binding energies comparable to those for ilomastat we then ran MD simulations to investigate the effect of dynamics on the Gibbs binding energies. For these MD simulations to reduce computational costs, the atoms in the first two environmental shells surrounding the ligand were free to move whereas the atoms in the third environmental shell and beyond were tethered. The whole protein-ligand complex was then solvated using a droplet boundary condition with a margin of 10. The waters were held in place by adding a potential wall with a weight of 100 to maintain the shape of the droplet. The system was minimised using the AMBER10:EHT FF to gradient of 0.1 kcal/mol Å^2^. Following the minimisation the systems were then ran for a 10 ns MD production simulation using the NPA algorithm. A time step of 2 fs was used. Light bonds were constrained and rigid waters were used.

### Glioma cell culture

The C6 glioma line (American Type Culture Collection, ATCC) maintains many characteristics of GBM including cancer stem cell behaviour ability to generate GBMs in rats upon injection in the brain^50–52^. The C6 clone stably expressing Cx43 demonstrates enhanced gap junctional intercellular communication, and restricted proliferation, with enhanced migratory potential (scrape-wound assay) when compared to wild-type C6^18^^,^^53^. Glioma cells were maintained at 37 °C, 95% air and 5% CO_2_ in a humidified incubator. Cells were cultivated in high glucose DMEM (4.0 g/L, ThermoFisher Scientific) containing L-glutamine (4.0 mM), antibiotics (penicillin, 100 IU/mL; streptomycin, 100 μg/mL) and 10% foetal bovine serum (FBS; Gibco). For collection of conditioned media, cells were plated in 6-well plate (200,000 cells/well) and grown to 80% confluency over the course of 3 days. At this time 3 ml of serum-free DMEM was added to each well and incubated for 24 h. For zymography, serum free medium was prepared with ilomastat (Selleck Chemical, S7157, purity >99.15%, 0, 25, 50, 75, or 100 μM). For NFF-3 experiments cells were grown to 80–90% confluence in 145 mm diameter plates, with DMEM was used for conditioning.

### Zymography

The conditioned media was concentrated using spin filters, with the tubes being centrifuged at 4800 g for 12 min in pre-chilled rotors. Protein concentration was determined by BCA assay and samples suspended in SDS loading buffer (no DTT and without boiling). Protein 100 µg/well were separated in 10% SDS-PAGE gels supplemented with 0.1% gelatine (125 V, ∼3 h). The gel was then washed twice in 45 min each in incubation buffer (50 mM Tris-HCl, pH = 7.5, 5 mM CaCl_2_, 1uM ZnCl_2_, 0.02% Brij-35, 0.02% NaN_3_, and 2.5% Triton X-100). The gel was washed in incubation buffer (50 mM Tris-HCl, pH = 7.5, 5 mM CaCl_2_, 1uM ZnCl_2_, 0.02% Brij-35 and 0.02% NaN_3_) which was replaced after 10 min. In a sealed container, gels were incubated at 37 °C for 20 h, followed by staining (Coomassie). Gels were processed in water to reveal the presence of activated MMPs as light bands against dark blue gel.

### SDS-PAGE/Western blotting

Conditioned media was collected and chilled on ice. Proteins were precipitated using cold acetone and chilled (−20 °C, 1 h). Proteins were pelleted by centrifugation (12 min, 4800 g). Proteins were suspended in SDS-PAGE loading buffer. A small amount of this material was diluted and taken for quantification by BCA. Protein samples were loaded on a 10% SDS-PAGE gel and separated under constant voltage (125 V). Proteins were then transferred at 30 V for 3 h at 4 °C, and blocked using 5% milk (TBST, 1 h). Membranes were incubated at 4 °C primary antibody (1:1000 rabbit antiMMP-3, ProteinTech, 1% milk in TBST) overnight. Membranes were washed 3x 15 min with TBST, followed HRPO-conjugated secondary antibody (1:2000, 1% milk, 1 h, room temperature). The membrane was washed three times with TBST (3x 15 min) prior to ECL imaging (Li-Cor, CDigit) and quantification (ImageJ).

### NFF-3 fluorescence assay

Conditioned media was then collected and distributed into a 96-well assay plate preloaded with 15 μL of a stock solution containing NFF-3 (100 μM in DMSO). Conditioned or control media (300 μL) was added to each well (final concentration NFF-3 = 4.76 μM, MW = 1675.8 g/mole) using a standard 96 well plate. MMP3 activity was monitored at 37 °C using a multi-well plate fluorimeter (SpectraMax M2, Molecular Devices). The proteolytic activity of C6-13 conditioned media was measured relative to the background fluorescence produced by unconditioned DMEM. Wavelengths for excitation (325 nm) and fluorescence emission (393 nm) were measured every 10 min over 6 h.The NFF-3 probe is from Cayman Chemical (395% pure, MW: 1675.8). After conditioning, the media was collected. Each experimental condition had its own control condition. Using a 96 well plate 15 μL of distilled water were added to the control wells and 15 μL of 100 μM NFF-3 were added to the experimental well. Then 300 μL of the conditioned media (C613, and DMEM media) were added to the control and experimental wells, resulting in an overall concentration of 4.76 μM NFF-3. The excitation wavelength of the spectrometer was set to 325 nm while the emission was set to 393 nm. The fluorescence was measured every ten minutes over the course of six hours, with the plate shaking for 15 s before each reading. The experiment was conducted at 37 °C.

## Results and discussion

### Inhibition of MMP3 in the conditioned media of C6-13 glioma

**Figure 2.**
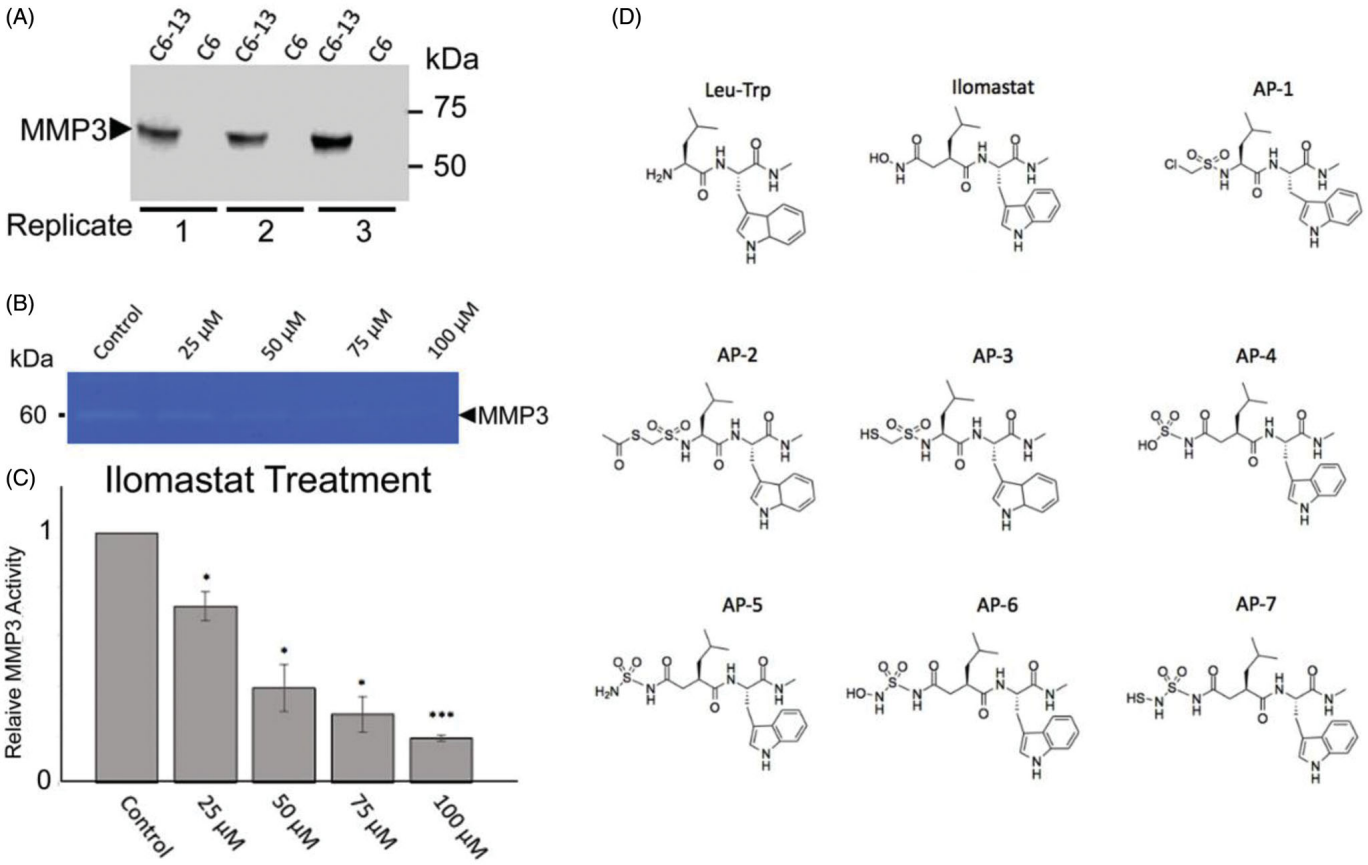
(A) Analysis of MMP3 in C6-Cx43 (C6-13) and low motility C6 parental line confirm MMP3 expression in high motility cells^18^^,^^53^. (B) Relative to untreated control, zymographic assays of C6-13 conditioned media demonstrate dose-dependent loss of MMP3 activity due to ilomastat (*N* = 3, significance level *p ≤ 0.05, ***p ≤ 0.001) (C). (D) Structures of the Leu-Trp backbone, ilomastat and sulphonamide derivatives computationally (Leu-Trp to AP-7) and experimentally (Leu-Trp, ilomastat and AP-1) examined in this study.

A recurrent in cancer is the observation that migration/invasion often coincides with the secretion of matricellular proteins and the activation of pathways that are typically reserved for normal development, tissue remodelling and repair. A key goal of GBM research has been to understand the underlying mechanisms of tumour infiltration. Cx43 promotes migration/invasion of glioma cells *in vitro* by wound healing, Transwell^TM^ assay, and brain slices; and *in vivo* using Cx43 wild-type and knockout mice^12^^,^^15^^,^^16^. Demonstrating a direct link to secreted factors and glioma motility, the transfer of C6-Cx43 conditioned media was found to be sufficient to stimulate movement of slower-moving cells^18^. Importantly, our previous proteomic analysis of the conditioned media of C6-13 cells demonstrated specific, significant increases in MMP3; with no other members of the MMP family was found to be differentially expressed between high and low motility cells^18^. In support of this view, we first performed SDS-PAGE/western blot analysis of MMP3 within the conditioned media from C6-Cx43, and C6 (parental line) serving as negative controls (Figure 2(A)). In biological triplicates, analysis of conditioned media revealed binary increases of MMP3 within the conditioned media of C6-Cx43 cells. Using zymographic assays incorporating natural substrate (0.1% gelatine), demonstrate the concentration-dependent inhibition of secreted MMP3 in glioma cells exposed to varying concentrations of ilomastat (0–100 uM). MMPs are secreted as inactive zymogens by the interaction of the zinc ion and the N-terminal (pro) domain. Removal of this domain by a collaborating enzyme initiates protease networks and degradative “cross-talk” to promote ECM remodelling^20^^,^^21^. Based on these activity profiles, we attributed the loss of MMP3 activity with the disruption of the protease-web. Cell death at ≤100 uM ilomastat was accessed (<1%) by flow cytometry (propidium iodide stain, data not shown).

### The docking of MMP inhibitors

Using ilomastat as a template several derivatives were modelled as potential inhibitors of MMP-3. Specifically, all ilomastat derivatives contain the same Leu-Trp backbone, but have different ZBGs (Figure 2(D)). For each compound shown in Figure 2 the respective top five scoring poses had very similar conformations. Therefore, for purposes of brevity and clarity only the top scoring conformer for each compound is discussed herein. For each compound docked in the active site of MMP3 the Zn^2+^ remained ligated by His201, His205 and His211 in agreement with the crystal structure^48^.

In the docking of ilomastat it was found that the top scoring pose had the alkoxide and carbonyl oxygen of the hydroxamic acid functional group ligating the Zn^2+^. The calculated distances of 1.913 Å and 1.707 Å for *r*(C=O…Zn^2+^) and *r*(O^−^…Zn^2+^), respectively indicate a strong interaction between the ZBG and the Zn centre. This bidentate binding of ilomastat to the Zn^2+^ results in the metal centre to have a trigonal bipyrimidal coordination geometry. Moreover, with the O^−^ atom of the hydroxamic acid ligated to the Zn centre Glu202 is able to act as a hydrogen bond donor to the hydroxamic acid alkoxide atom where a ^Glu202^O…O^−^ distance of 2.815 Å was calculated. Lastly, the bidentate ligation of ilomastat to Zn^2+^ allows the amide group of the hydroxamic acid to hydrogen bond to the backbone carbonyl of Ala165 where *r*(^Ala165^C=O…N) was calculated to be 2.971 Å. As discussed by Jacobsen et al. ^49^, such bonding/interaction between hydroxamic acids and MMP3 is crucial.

Regarding the ligands not containing acidic protons, (i.e. **Leu-Trp**, **AP-1**, **AP-2**, and **AP-5**) none of the ligands had the proposed ZBGs (Table 1) bind to the Zn^2+^. In the case of **Leu-Trp**, **AP-1**, and **AP-2**, the interaction with the Zn^2+^ ion occurred via the carbonyl groups in the Leu-Trp backbone. Specifically, **Leu-Trp** ligated the Zn^2+^ via the Trp and Leu carbonyl O-atoms where *r*(^Trp^C=O…Zn) and *r*(^Trp^C=O…Zn) were calculated to be 2.092 Å and 2.197 Å, respectively. In the case of **AP-1** it was found to only ligate the Zn^2+^ via the carbonyl oxygen atom of Leu where *r*(^Leu^C=O…Zn) =2.228 Å. Similarly, for **AP-2** it was found to only monodentately ligate the Zn^2+^, however, it was via the Trp carbonyl O-atom where *r*(^Trp^C=O…Zn) = 2.171 Å. Regarding **AP-5**, it was found that an oxygen from the sulphonamide coordinated to the Zn^2+^ ion where *r*(S = O…Zn) = 2.006 Å where the ZBG amine did not ligate the Zn^2+^ centre. For the top scoring poses of **Leu-Trp**, **AP-1**, **AP-2**, and **AP-5** no hydrogen bonding interaction was seen between Glu202 and the ZBG. Moreover, no hydrogen bonding interaction between the backbone carbonyl of Ala165 and the ZBG was observed.

For the ionisable ligands **(i.e. AP-3**, **AP-4**, **AP-6**, and **AP-7**) all were found to have the ZBG ligate the Zn centre. For **AP-3**, the ZBG was found to monodentately ligate the Zn^2+^ ion via the anionic thiolate where *r*(S^−^…Zn^2+^) was calculated to be 2.162 Å. However, it was found that the ^Glu202^CO_2_^−^…S distance was 4.183 Å. Given the considerable length, it is unlikely that a suitable H-bond interaction would exist between Glu202 and the ZBG of **AP-3**, to enhance the binding of the ligand. Regarding the possible hydrogen bond to Ala165 no H-bonding interaction was observed for **AP-3**. For **AP-4** the sulphate was found to form a bidentate interaction where two of the oxygen atoms of the sulphate were coordinated to Zn^2+^. Specifically, **AP-4** binds the Zn^2+^ is a germinal type binding where the S = O…Zn^2+^ distances were calculated to be 2.031 Å and 2.671 Å. It is noted that the ability of the phosphinate functional group to reproduce the gem-diol intermediate of the transition state during peptide hydrolysis has been investigated as possible ZBGs of MMPIs^29^^,^^43^. The ^Glu202^CO_2_^−^…O = S distance was calculated to be 2.895 Å indicating the presence of a H-bonding interaction. Regarding the possible hydrogen bond to Ala165 no H-bonding interaction was observed for **AP-4**.

In the docking of **AP-6** and **AP-7** it was found that both ligands bidentately ligate the Zn^2+^. However, unlike ilomastat where the carbonyl and the alkoxide oxygen atoms of the hydroxamic acid functional group ligate the Zn^2+^ it was found that for **AP-6** and **AP-7** the bidentate ligation occurs through the O^−^ and N atom of **AP-6** and the S^−^ and N atoms of **AP-7** via a side-on type interaction. This difference between ilomastat and **AP-6**/**AP-7** is likely due to the presence of the sulphonyl functional group that results in the amine not being planar which allows the lone pair to engage in bonding to the Zn^2+^ ion via a side-on interaction. In the case of **AP-6**, the calculated *r*(N…Zn^2+^) and *r*(O^−^…Zn^2+^) distances were calculated to be 2.017 Å and 1.966 Å, respectively indicating a strong interaction with the Zn^2+^ ion. Concerning Glu202 it was found that *r*(^Glu^_202_CO_2_…O^−^) = 2.891 Å indicating a strong H-bonding interaction with **AP-6**. Similarly, for **AP-7**, a strong interaction with the Zn^2+^ ion exists where the calculated *r*(N…Zn^2+^) and *r*(S^−^…Zn^2+^) distances were 2.124 Å and 2.267 Å, respectively. However, regarding Glu202 a weak H-bond interaction exists where *r*(^Glu202^CO_2_…S) was calculated to be 3.200 Å.

**Figure 3.**
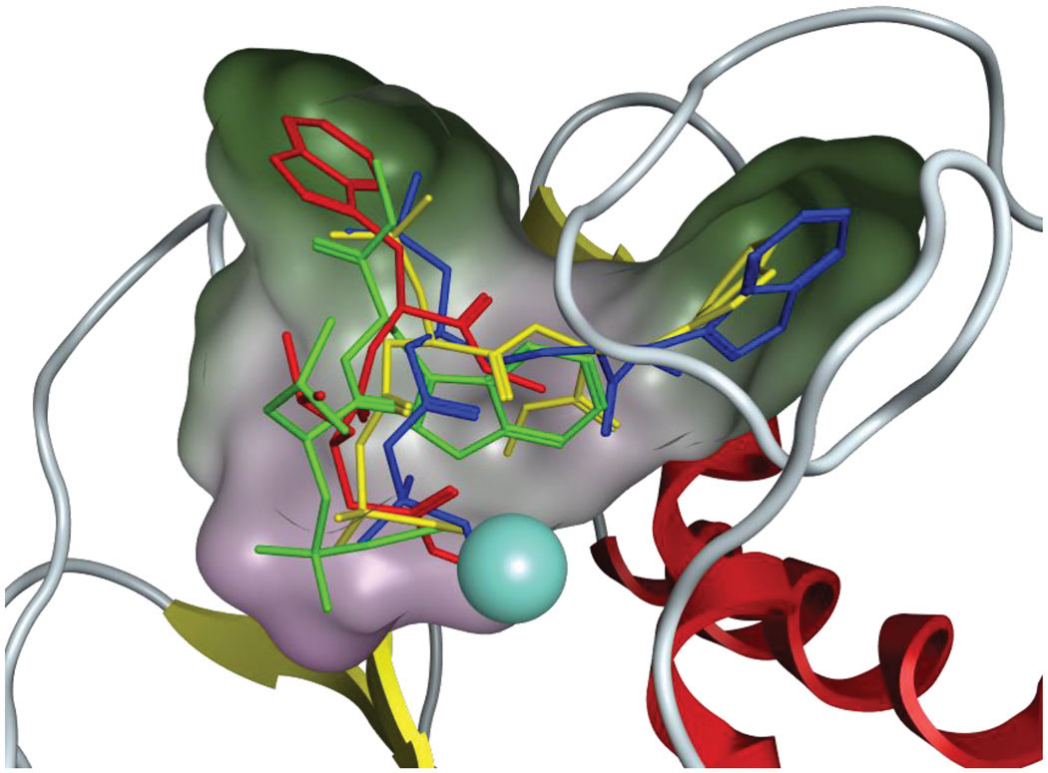
The placement of **AP-3**, **AP-6**, **AP-7** and ilomastat in MMP3. The colour scheme is green=**AP-3**, blue=**AP-6**, yellow=**AP-7**, and red = ilomastat. Regarding the molecular surface green indicates lipophilic regions and purple indicates low lipophilic regions. The Zn^2+^ ion is represented by the large light blue coloured sphere.

Figure 3 shows the placement of **AP-3**, **AP-6**, **AP-7** and ilomastat in the binding site of MMP3. From Table 1 **AP-3**, **AP-6**, **AP-7** and ilomastat all had predicted Gibbs binding energies more negative than −10 kcal mol^−1^. In all cases the ZBG of each compound is ligated to the Zn^2+^ ion, however the longer ZBG of **AP-6** and **AP-7** results in a different binding mode between ligand and MMP3 than seen for ilomastat. From Figure 3 the imidazole functional group of ilomastat is located in a lipophilic region which is a favourable interaction. For **AP-6** and **AP-7** the leucine side chain is, however, located in this region. For **AP-6** and **AP-7** the imidazole ring is instead located in a different lipophilic region. In the case of **AP-3** it can be seen that neither the imidazole nor the leucine side chain is located in a lipophilic region providing some understanding of its weaker Gibbs binding energy (Table 1). Figures S3 and S4 show another perspective of the binding of **AP-3**, **AP-6**, **AP-7** and ilomastat to MMP3.

The calculated Gibbs binding energies of the compounds tested are given in Table 1. From Table 1, it can be seen that in general the presence of an acidic proton resulted in the Gibbs binding energies being more negative than those ligands without acidic protons. The exception being **AP-4**. Moreover, those ligands that formed a hydrogen bonding interaction with Glu202 resulted in increased Gibbs binding energy between MMP3 and ligand (**AP-3**, **AP-6,** and **AP-7**). Such results are agreement to the work by Jacobsen et al.^49^ From the Gibbs binding energies provided in Table 1 the compound **AP-6** was found to have a more negative Gibbs binding energy than ilomastat suggesting that it would be a stronger inhibitor. However, it is noted that for **AP-7** the calculated Gibbs binding energy was only 0.5 kcal mol^−1^ less negative than ilomastat. Thus, the bidentate ligation by the anionic binding group is key to strongly binding to the Zn^2+^. Regarding **AP-3** even though the ligand only ligates the Zn^2+^ ion through the S^−^-atom it still has a relatively large negative Gibbs binding energy of −11.2 kcal mol^−1^. As discussed above the poorer binding energy of **AP-3** is likely due to unfavourable placement of the side chains of backbone Trp and Leu residues.

### The synthesis of the MMP inhibitors and NFF3 analysis

**Figure 4.**
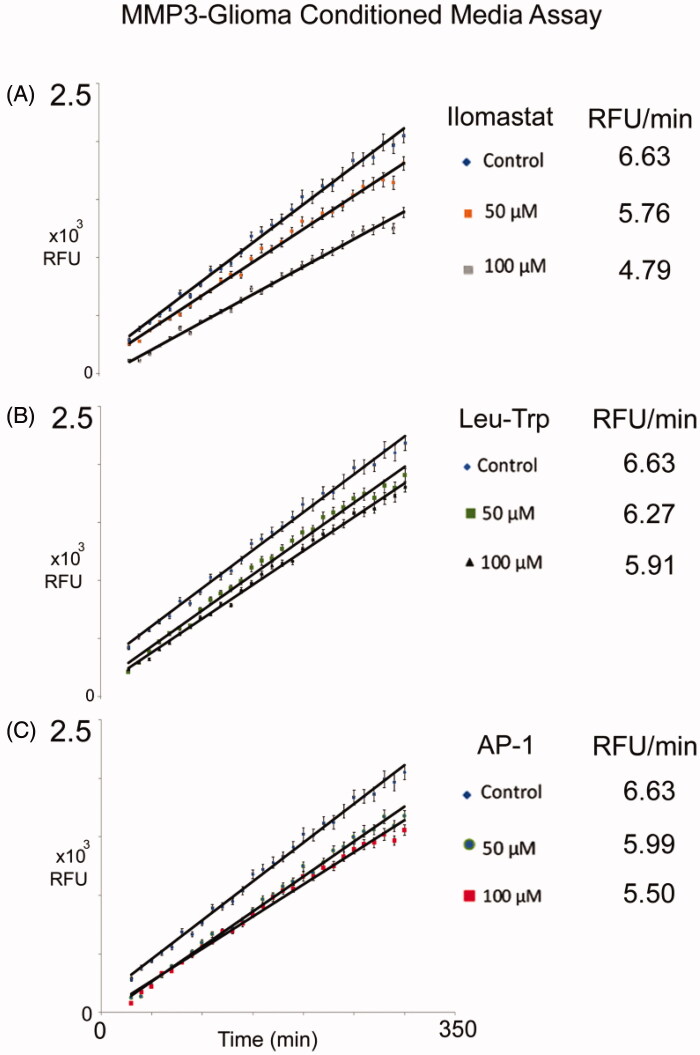
Inhibition of MMP-3 activity detected by NFF-3 assay. Assays were monitored over six hours, relative to untreated controls, 50 μM and 100 μM concentration for each of the listed compounds were compared for: (A) Ilomastat, (B) Leu-Trp, and (C) AP-1. Value reported here represent average, control normalised values performed in triplicate (*N* = 3), *R*^2^ values >0.99. Error bars representing standard error of the mean.

The leucine-tryptophan (**Leu-Trp**) backbone, and **AP-1** were synthesised. All synthesised compounds were analysed by mass spectrometry (Figure S2). The NFF-3 assay was used to determine the biological inhibition of MMP-3 activity for ilomastat and synthesised compounds, **Leu-Trp**, and **AP-1** relative to untreated controls. NFF-3 is a substrate selective for MMP3 (kcat/Km = 218,000 s^−1^ M^−1^), and to a much lesser degree, MMP9 (kcat/Km = 10,000 s^−1^ M^−1^)^54^. Consistent with our MMP3 SDS-PAGE/westernblot datum, the conditioned medium of C6-Cx43 demonstrated robust fluorescence activity compared to low motility C6 cells and unconditioned DMEM^18^. Linear responses of MMP3 activity was robustly measured from 30 to 300 min. All compounds were tested at a concentration of 50 μM and 100 μM, and compared to determine MMP3 suppression. From Figure 4 concentration dependent inhibition was observed. Here it can be seen that for all compounds the 100 μM was better at inhibiting MMP-3 than the 50 μM concentration. This analysis also demonstrates Leu-Trp backbone can act as a competitive inhibitor. Consistent with this interpretation, as discussed above in the docking study, **Leu-Trp** was found to ligate the Zn^2+^ via the Trp and Leu backbone carbonyl O-atoms. Demonstrating the relative contributions of the ZBG in **AP-1** and ilomastat, both compounds perform better than **Leu-Trp** at inhibiting MMP-3.

**Figure 5.**
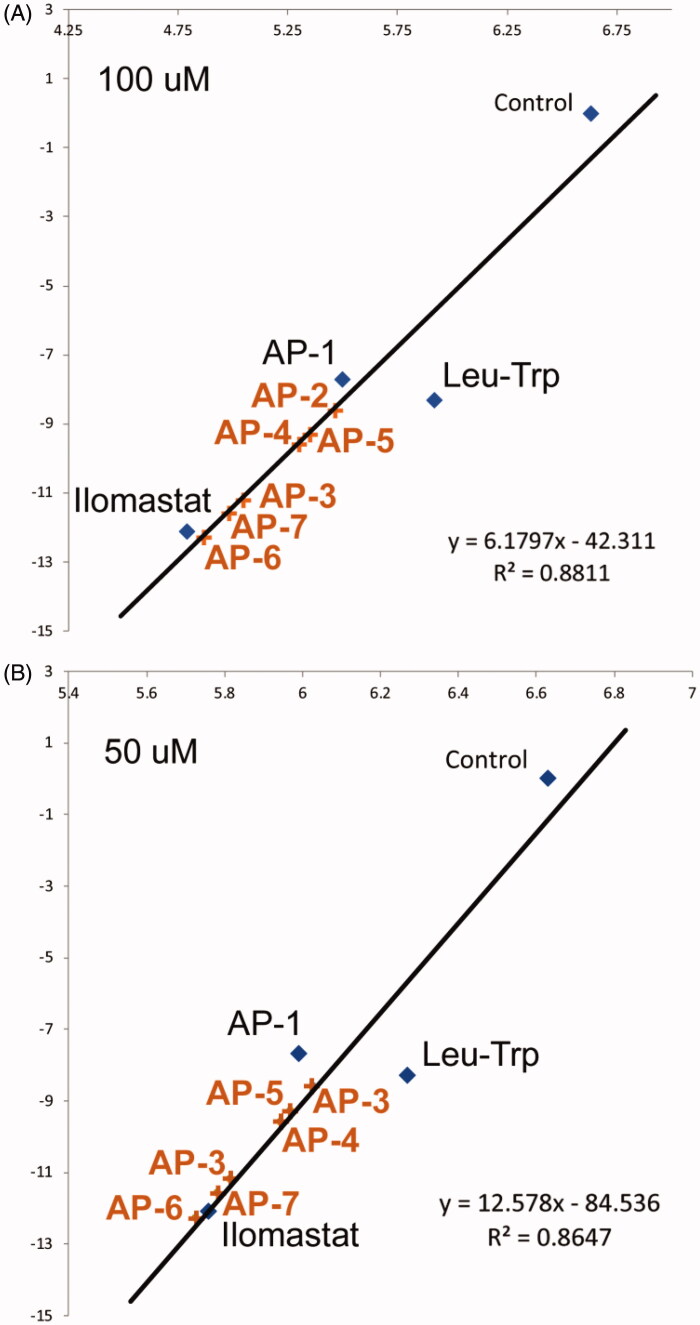
Comparison of MMP3 activity (NFF-3 fluoresence, RFU/min) in the presence of **Leu-Trp**, **AP-1**, ilomastat at (A) 50 μM and (B) 100 μM and negative control as a function of calculated binding affinity. Anticipated inhibitory performance of (**AP-2**, **AP-3**, **AP-4**, **AP-5**, **AP-6** and **AP-7**) are based on linear regression models.

Binding energy scores provide valuable input to predict the performance of our sulphonamide-based MMPIs in biological matrices relevant to invasive GBM. The change in RFU value for each compound was normalised to the control, resulting in a value of less than one. Normalised values were plotted against overall binding energy (Figure 5). A line of best fit was generated with an R^2^ value of 0.8647 for the inhibitors at 50 μM, and 0.8811 for inhibitors at 100 μM. This indicates a good correlation between the experimental and computational results. Inhibitory potential for the remaining compounds not synthesised are included within the graph (Figure 5(a,b)), based on calculated values using the following relationship:

(1) $${\text{MMP}3\;\text{Activity}}_{50\text{uM}}=\frac{\text{Binding}\;\text{Affinity}\;\pm \;84.536}{12.578}$$

(2) $${\text{MMP3 Activity}}_{100\text{uM}}=\frac{\text{Binding Affinity}\pm 42.311}{6.1797}$$

From Figure 4 the compounds **AP-3, AP-6 and AP-7** are predicted to have similar or better biological performance than ilomastat. This suggests these compounds worth examining to determine the precise mechanisms of interaction using MD simulations.

### Refinement of the binding affinities

Using the top scoring docking pose for ilomastat, **AP-3**, **AP-6**, and **AP-7** we ran 10 ns MD simulations (see Methods for more detail) to investigate the effect of protein dynamics on the inhibitory effects of ilomastat, **AP-3**, **AP-6**, and **AP-7**. Using the trajectories from the MD simulations the Gibbs binding energy for each snapshot from the simulation was calculated using the MD_Analysis tool in MOE. Specifically, the binding energy was calculated using the GBVI/WSA dG scoring function. The results of which have been averaged (with standard deviations provided) for ilomastat, **AP-3**, **AP-6**, and **AP-7** and are given in Table 2.

**Table 2.** Average lengths for key bonds between ligand and MMP3. Average Gibbs binding energies with standard deviations.

Model	O – Zn (Å)	N – Zn (Å)	Glu202CO2…O (Å)	ΔbindG (kcal mol^−1^)
**AP-3**	1.908 ± 0.032	N/A	2.990 ± 0.142	−10.8 ± 0.3
**AP-6**	1.728 ± 0.032	1.847 ± 0.040	2.645 ± 0.102	−13.7 ± 0.4
**AP-7**	1.964 ± 0.377^b^	1.962 ± 0.071	2.921 ± 0.107^c^	−13.3 ± 0.4
**Ilomastat**	1.717 ± 0.030	1.966 ± 0.091^a^	2.653 ± 0.102	−13.1 ± 0.4

^a^ In the case of ilomastat the coordinating atom was the carbonyl oxygen of the hydroxamic acid functional group and not the nitrogen as seen for **AP-6** and **AP-7**.

^b^ The coordinating atom was the sulphur of **AP-7**.

^c^ The average distance is for the **^Glu202^CO_2_…S** interaction.

From the values in Table 2 it can be seen that in general the binding energies become more negative indicating a stronger binding to MMP3. The exception being **AP-3** where the binding energy has become marginally less negative. Notably, from the values provided in Table 2 **AP-6** and **AP-7** are predicted to bind to MMP-3 stronger than ilomastat. Specifically, for ilomastat Δ_bind_G = −13.1 ± 0.4 kcal mol^−1^, whereas for **AP-6** and **AP-7** Δ_bind_G was calculated to be −13.7 ± 0.4 kcal mol^−1^ and −13.4 ± 0.4 kcal mol^−1^, respectively.

From Table 2 it can be seen that for ilomastat, **AP-3**, **AP-6**, and **AP-7** the compounds remain strongly ligated to the Zn^2+^ ion. Moreover, from the simulations it can be see that for all four compounds the active site Glu202 interaction has become stronger given the shorter average ^Glu202^CO_2_…O distance than seen in the docking simulation. Regarding the involvement of Ala165 in the binding of ilomastat, **AP-3**, **AP-6**, and **AP-7** it was found that from the MD simulation ^Ala165^C=O…*N* = 2.925 Å ± 0.188 Å for ilomastat. For **AP-3** the ^Ala165^C=O…N distance was calculated to be 4.384 Å ± 0.309 Å whereas for **AP-6** the ^Ala165^C=O…N distance was calculated to be 4.384 Å ± 0.309 Å. In the case of **AP-7** the ^Ala165^C=O…N distance was calculated to be 4.343 Å ± 0.294 Å. Thus, based on the compounds investigated it appears that the presence of the hydroxamic acid is essential for Ala165 to form a H-bonding interaction with the ligands. However, from the results it appears that the presence of an anionic ZBG that bidentately ligates the Zn^2+^ ion as well as the ability to form a hydrogen bond to Glu202 is more crucial for the development of potent MMP3 inhibitors whereas the interaction with Ala165 is not predicted to be critical to the inhibition of MPP-3 given the binding energies in Table 2. As noted in the introduction a failure of present MMPIs is poor transition metal/MMP selectively^35^. As a means to improve selectivity ligands have been designed with thiol groups rather than hydroxyl groups as a way to make inhibitors that are selective to the zinc containing MMPs^29^^,^^55–61^. Thus, while **AP-6** and **AP-7** are both predicted to bind to MMP3 the presence of the thiol in **AP-7** may enhance the selectivity of **AP-7** to the Zn^2+^ containing MMPs versus non-Zn containing metalloproteins.

## Conclusions

As a barrier for cancer invasion, the extracellular environment has received much attention to identify avenues to inhibit cancers, including GBM^62^. To facilitate invasion, cancer cells modify their secretion to alter the ECM. Increases in glioma migration are accompanied by the secretion of regulatory proteins, such as MMPs. A key goal of GBM research has been to understand the microenvironment of tumour cells, to develop therapeutic strategies to limit invasion. Previous studies examining actively migrating glioma cells have demonstrated mutually exclusive mechanisms regulating invasion and cell proliferation^63^^,^^64^. Appearing to engage these mechanisms, the gap junction protein Cx43 in glioma has demonstrated a strong effect on the suppression of tumour growth^65–67^ and promotes migration and invasion of cells^12–15^. Providing the experimental framework to better understand pathways available to invasive glioma, we previously conducted system-wide analysis to identify proteins that are differentially expressed by high/low motility C6 cells^18^. Demonstrating the selective engagement of ECM remodelling networks, data presented here confirm the binary increase, activity and inhibition of MMP3 using the condition media of glioma cells. As invasive behaviour is a hallmark of GBM, we were particularly interested MMPs secreted by high motility glioma cells as a target for therapeutic design.

In the present work ilomastat was used as a template to develop derivatives to inhibit MMP3. For ilomastat and synthesised compounds, NFF-3 and zymography assays showed a significant correlation between the experimental and theoretical results. Linear regression models provided may be used for the predict in silico performance of future MMPIs at 50 and 100 uM, (Equation (1) and (2) within a complex matrix, representing the environment and MMP3 expressed by high motility C6 cells.

In addition to the experimental work several ilomastat derivatives were computationally investigated to evaluate their potential as MMPIs. All derivatives contained the same backbone, but with different ZBGs. Specifically, all analogues contained a sulphonamide ZBG. Ilomastat and its derivatives were docked and scored, generating their relative Gibbs free binding energies. The compounds with the greatest binding affinities, ilomastat, **AP-6**, and **AP-7**, all showed bidentate coordination with the Zn^2+^ ion. Moreover, these three compounds formed strong hydrogen bonding interactions with Glu202. It is noted that such an interaction has been previously deemed to be important in binding. To investigate the effect of protein dynamics on the inhibitory effects of the strongest binding compounds 10 ns MD simulations were ran. From the MD simulations, **AP-6** and **AP-7** showed a stronger Gibbs binding affinity than ilomastat. Therefore, from this study it can be concluded that **AP-6** and **AP-7** show promising results as potential broad-spectrum inhibitors of MMPs. However, the selectivity of **AP-7** to the Zn^2+^ containing MMPs versus non-zinc containing metalloproteins may be greater than **AP-6** or ilomastat given the presence of the thiol in **AP-7**. Synthesis of **AP-6** and **AP-7** are currently underway to determine the effect on migration and invasion.

## Supplementary Material

Supplemental Material
