# Supplementary material for: Examination of sulfonamide-based inhibitors of MMP3 using the conditioned media of invasive glioma cells
Source: J Enzyme Inhib Med Chem. 2020 Mar 11;35(1):672–81. doi: 10.1080/14756366.2020.1715387 (PMC7144313; doi:10.1080/14756366.2020.1715387)
Supplement: Supplemental Material [file IENZ_A_1715387_SM3974.pdf]

Computational Examination of Sulphonamide-based MMP Inhibitors Modelled Using the Conditioned Media of Invasive Glioma Cells

Alisha T. Poole[‡1]*, Christopher A. Sitko*[‡1]*, Caitlin Le*[1]*, Christian C. Naus*[2]*, Bryan M. Hill*[1]*, Eric A. C. Bushnell*[1*]*, and Vincent C. Chen*[1]*

[1] Department of Chemistry, Brandon University, 270-18th Street, Brandon, Manitoba R7A 6A9, Canada

[2] University of British Columbia, Life Science Institute, 2350 Health Science Mall, Vancouver, British Columbia, V6T 1Z3, Canada

AUTHORS EMAIL ADDRESS: bushnelle@brandonu.ca and chenv@brandonu.ca

RECEIVED DATE: to be inserted after manuscript is accepted

‡ Authors contributed equally to this work.

* Authors to whom correspondence should be addressed. Email: bushnelle@brandonu.ca and chenv@brandonu.ca

Supplementary Information

4 Figures, 5 Pages in total.

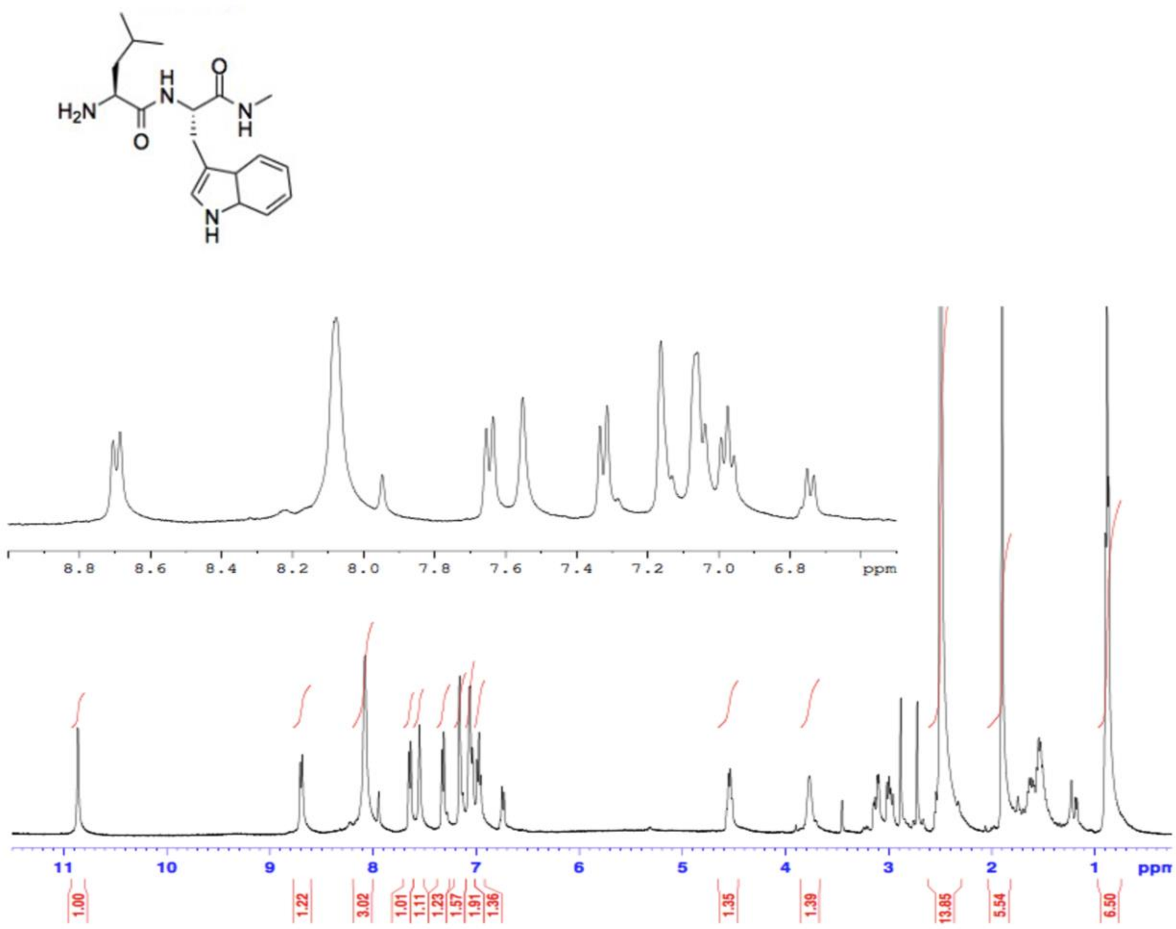

Figure S1: ^{1}H-NMR of tryptophan-leucine backbone. The peak at ~11 ppm corresponds to the H on the N of the Trp five carbon ring. The peaks around ~7 ppm correspond to Trp aromatic H. The peak just before ~1 ppm corresponds to the H on the methyl groups of leucine.

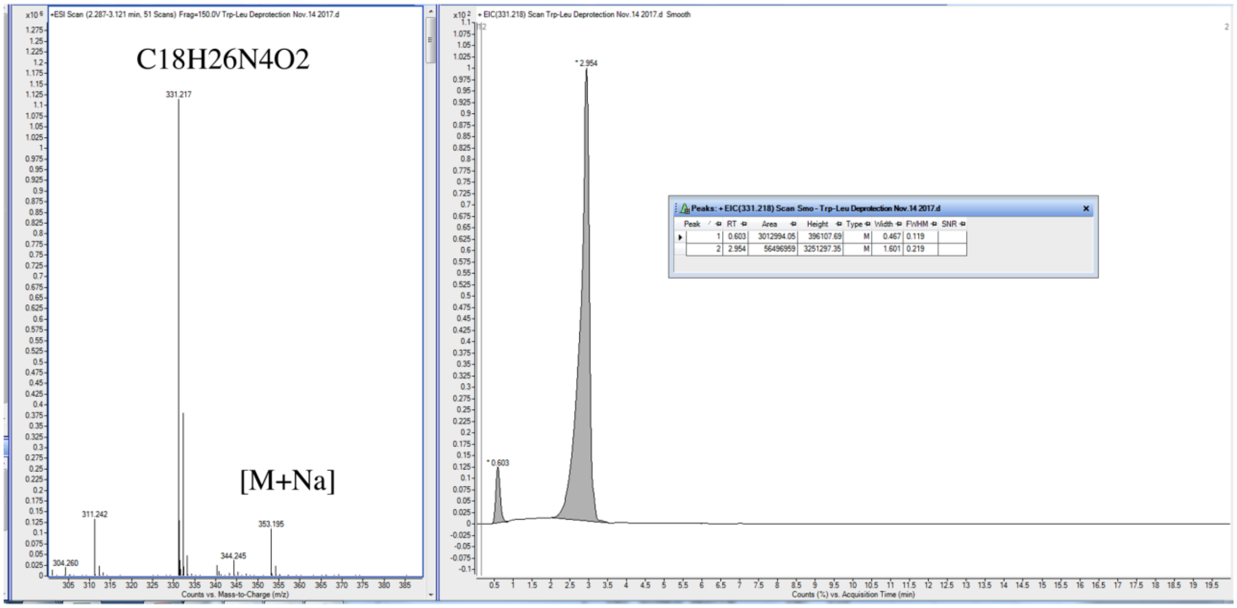

a)

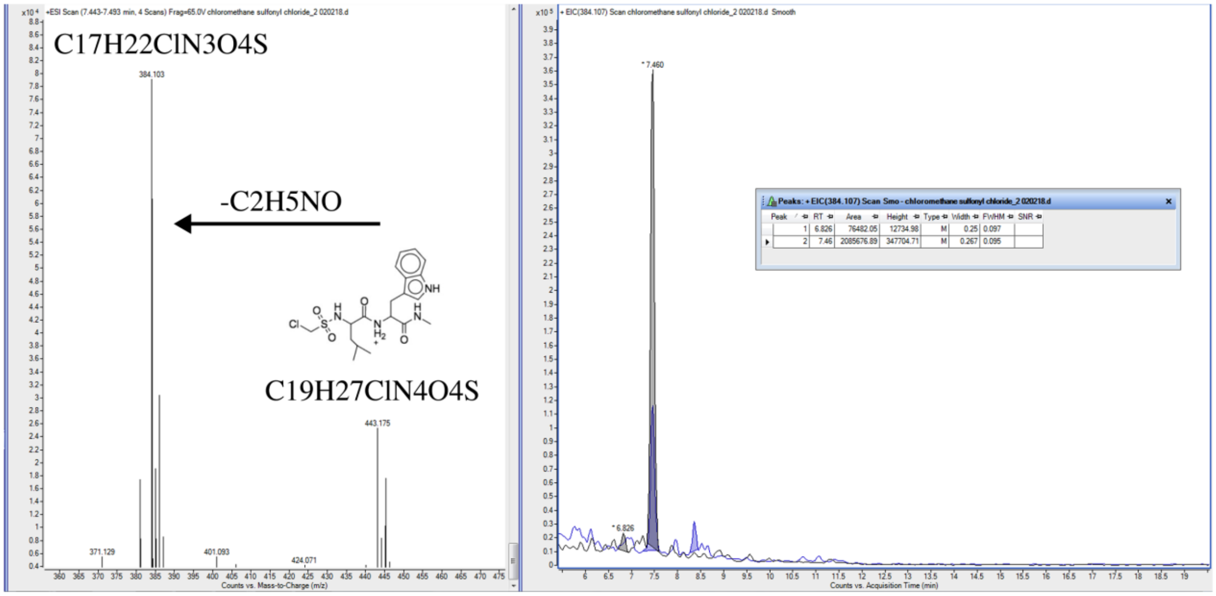

b)

Figure S2. Mass spectrometry results for (**a**) Leu-Trp and (**b**) AP-1. Experimental M+H values located under each spectra correspond to the peaks determined by mass spectrometry for each compound.

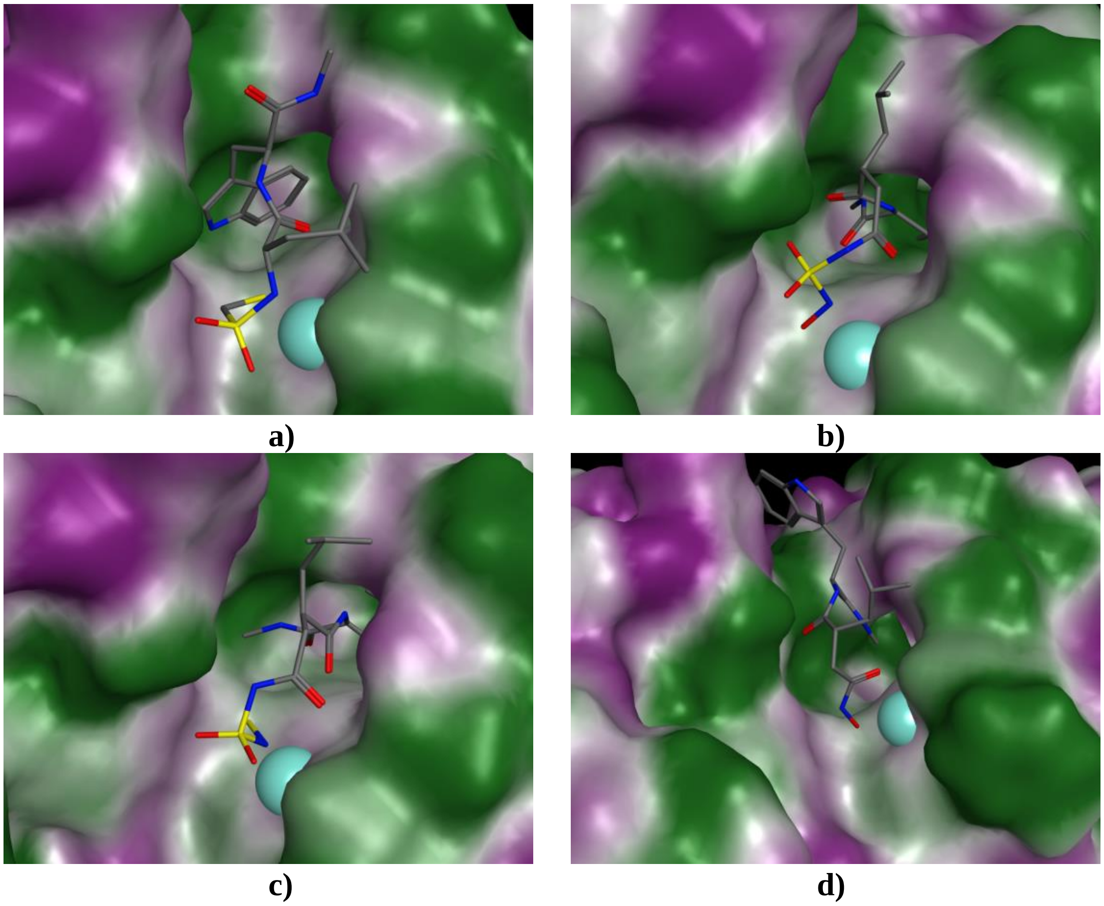

Figure S3. The binding of a) **AP-3**, b) **AP-6**, c) **AP-7** and d) ilomastat to MPP3. The Zn^{2+} ion is represented by the large light blue coloured sphere. Regarding the molecular surface green indicates lipophilic regions and purple indicates low lipophilic regions.

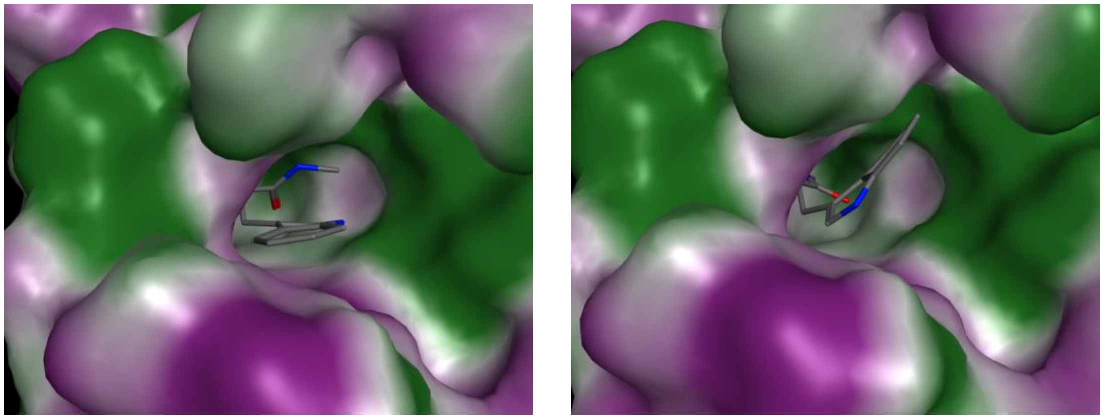

a) b)

Figure S4. The binding of the imidazole rings of **a**) **AP-6**, and **b**) **AP-7** to MPP3. Regarding the molecular surface green indicates lipophilic regions and purple indicates low lipophilic regions.
